# Hernia Surgery Beyond Technique—A Discipline Redefining Itself

**DOI:** 10.3390/jcm15083031

**Published:** 2026-04-16

**Authors:** Jacob Rosenberg

**Affiliations:** Department of Surgery, Herlev Hospital, University of Copenhagen, DK-2730 Herlev, Denmark; jacob.rosenberg@regionh.dk

As this Special Issue of the *Journal of Clinical Medicine* reaches its conclusion, it is becoming increasingly evident that hernia surgery—long seen as one of the most standardized and technically mature domains of general surgery—is undergoing a profound conceptual transformation [1]. For decades, progress in hernia surgery has been linked to technical improvements: better meshes, enhanced fixation techniques, the shift from open to laparoscopic repair, and, more recently, the rise of robotic platforms. These advancements have clearly improved results, particularly by lowering recurrence rates and perioperative complications. However, ironically, the success of these innovations has revealed the limitations of relying solely on a technical approach. The contributions in this Special Issue show that hernia surgery is no longer mainly about how we perform the operation. Instead, it emphasizes why, when, and for whom we operate, along with, perhaps most importantly, how we define success.

Modern hernia surgery is a remarkable technological success story. Recurrence rates, once the main measure of quality, have dropped significantly since the adoption of mesh-based repair techniques [2,3,4,5]. Minimally invasive methods have further decreased postoperative pain and accelerated recovery [6,7,8]. And yet dissatisfaction persists. The persistence of chronic postoperative pain, functional impairment, and patient dissatisfaction challenges the idea that technical success equals clinical success. Chronic postoperative inguinal pain (CPIP), in particular, has become one of the most significant complications in modern hernia surgery, impacting a notable minority of patients [9,10,11,12].

This gap between surgeon-defined success and patient-experienced outcomes is not unique to hernia surgery, but it is especially noticeable in this domain. Hernia repair is mainly performed to improve quality of life rather than prevent death. When such improvement is not achieved—or is offset by new symptoms—the entire rationale for intervention is questioned. The perspective article included in this Special Issue [13] clearly states that patient-reported outcomes (PROs) must move from the periphery to the center of surgical evaluation. This is not just a minor methodological adjustment; it represents a complete redefinition of the goals of surgery.

If recurrence characterized the pre-mesh era, chronic pain defines the present. The corresponding review in this Special Issue highlights the heterogeneity and complexity of CPIP [14], with reported incidence rates varying widely across methodologies, follow-up durations, and surgical techniques. This variability highlights deeper issues in the literature. Definitions vary, outcome measures differ, and validated instruments are not always used. As a result, accurately quantifying the true burden of chronic pain remains challenging [12]. More importantly, the field has yet to fully embrace prevention.

The pathophysiology of CPIP is multifactorial. Neuropathic pain may result from nerve injury or entrapment, while nociceptive pain may be related to mesh-induced inflammation or tissue remodeling. Patient-related factors—including psychological profiles, pain sensitivity, and expectations—also play a critical role. Despite this complexity, the current approach remains largely reactive, focusing on treatment rather than prevention. A more forward-looking strategy would integrate risk stratification into surgical decision-making, identify high-risk patients preoperatively, and adjust techniques accordingly. In this sense, chronic pain reveals a fundamental limitation of a purely technical approach: the outcome is not determined solely in the operating room.

While elective surgery has benefited from decades of refinement, emergency hernia surgery still carries significant risks of morbidity and mortality. The nationwide Swedish register study included in this Special Issue confirms that emergency groin hernia repair carries significantly higher risks than elective repair, including increased mortality, complications, and the need for bowel resection [15]. These findings align with earlier registry-based studies and emphasize a persistent challenge in surgical care [16,17,18]. What is striking is not the existence of this problem but its persistence.

Emergency hernia surgery often indicates a failure in regard to timing. It results from delayed diagnosis, referral, or decision-making. While watchful waiting has been proven safe for certain patients [19,20], its use must be carefully considered. The data presented here suggest that risk stratification should play a more central role in decision-making. Patients with femoral hernias, of advanced age, or with significant comorbidities may benefit from earlier elective intervention, even in the absence of severe symptoms. This represents a shift from a symptom-based to a risk-focused model of care.

Comparison of eTEP and open sublay repair shows how surgical techniques for ventral hernia repair continue to evolve [21]. Minimally invasive techniques continue to develop, a process driven by the benefits of fewer complications and quicker recovery. The eTEP method exemplifies this trend, providing a minimally invasive approach that maintains the advantages of a retromuscular mesh position. The reported improvements in postoperative outcomes are promising. However, they also raise important questions. Much of the evidence supporting new techniques comes from single-center studies or highly specialized surgeons. Issues like reproducibility, learning curves, and long-term outcomes remain only partially understood. This issue is not unique to hernia surgery. In many surgical fields, innovation often outpaces proper evaluation. The challenge ahead consist of matching the speed of innovation with thorough evidence collection. This endeavor will require multicenter collaboration, standardized outcome reporting, and long-term follow-ups—elements that have historically been underdeveloped in surgical research.

The study on incisional hernia after ileostomy closure [22] reveals a troubling fact: many hernias are directly caused by surgical procedures. Incisional hernias continue to be common following abdominal surgery, significantly affecting patients’ quality of life and healthcare resources. Recognizing obesity and previous hernia as independent risk factors aligns with existing research, but the implications transcend simple risk assessment. They challenge us to rethink the role of surgery. If hernias are partly iatrogenic, then prevention becomes a key responsibility. This includes optimizing patient conditions—addressing modifiable risk factors such as obesity, smoking, and malnutrition—as well as employing technical strategies, including prophylactic mesh placement. Randomized trials have shown that a prophylactic mesh can significantly lower incisional hernia rates in high-risk patients without introducing major complications [23,24]. A central guideline from 2022 recommends the use of a prophylactic mesh to reduce the occurrence of incisional hernias in at-risk patients, stressing that the mesh should be placed as an onlay or in the retromuscular plane [25]. Despite this, adoption remains inconsistent. This reluctance stems from both practical concerns and conceptual inertia. Preventive strategies demand a shift in mindset, moving from managing complications to preventing them.

One of the less visible yet increasingly important developments in hernia surgery is the growing emphasis on perioperative optimization. Hernia repair has often been perceived as a low-risk, routine procedure requiring minimal preparation. However, this perception is becoming increasingly outdated. Patients undergoing hernia surgery are now typically older, have more comorbidities, and present with more complex health profiles than in previous decades. Optimization strategies—including nutritional support, prehabilitation, smoking cessation, and glycemic control—have been shown to improve outcomes in other surgical areas. Their application to hernia surgery makes sense but is still inconsistently implemented. The integration of perioperative optimization into standard care presents an opportunity to improve results beyond what technical improvements alone can deliver.

The observational study on tailored management strategies [26] in this Special Issue reinforces a central theme: there is no one-size-fits-all approach to hernia repair. Patients vary in important ways. Age, comorbidities, anatomy, previous surgeries, and personal preferences all influence the best choice of technique and anesthesia. Guidelines offer helpful frameworks, but they cannot account for all the complexities of clinical practice. The shift toward personalized surgery reflects a broader trend in medicine that emphasizes individualized decision-making. However, personalized care also introduces challenges for research. Standardization makes comparisons easier, but it can hide important differences among patients. Researchers must find ways to balance these competing needs, possibly through registry-based studies and pragmatic trial designs.

A recurring theme across the contributions in this Special Issue is the significance of high-quality data. National hernia registries, especially in Scandinavia, have played a pivotal role in advancing the field. They offer large-scale, real-world data that complement randomized trials and support long-term follow-ups. Incorporating patient-reported outcomes into these registries is an important next step. By merging clinical data with patient-centered information, registries can provide a more complete view of surgical results. In the digital health age, there is also an opportunity to utilize new technologies for data collection, such as mobile health apps and remote monitoring. These tools may allow outcome assessments, especially those that are important to patients, to be conducted more often and in greater detail.

The most profound shift highlighted in this Special Issue is the redefinition of surgical success. Traditional metrics—recurrence, complications, and length of stay—are important, but they are not enough. In benign conditions, the main goal is to improve quality of life. The perspective article included in this issue argues that PROs must be central to outcome evaluation [13]. This notion is backed by a growing body of literature emphasizing the importance of quality of life, functional recovery, and patient satisfaction [27,28]. This shift has implications at multiple levels. At the research level, it requires incorporating validated PRO tools into clinical trials. At the clinical level, it requires engagement with patients as partners in decision-making. At the system level, it calls for aligning quality metrics and reimbursement models with outcomes that matter to patients.

Taken together, the contributions in this Special Issue indicate that hernia surgery is undergoing a fundamental transformation. It is moving from technical optimization to focusing on outcomes, standardized protocols to personalized care, surgeon-centered metrics to patient-centered evaluation, reactive treatment to proactive prevention, and isolated procedures to comprehensive perioperative pathways. This shift reflects broader trends in medicine, but it is especially evident in hernia surgery, where the main goal is to improve quality of life.

I want to sincerely thank all the authors who contributed to this Special Issue, the reviewers for their thoughtful and constructive feedback, and the editorial team for their support throughout the process. The work presented here is not an endpoint: it is a transition. The future of hernia surgery will not be defined solely by the perfection of technique but by our ability to match surgical practice with the outcomes that matter most to patients. The question is no longer whether we can repair a hernia but whether we can do so in a way that truly improves the life of the afflicted individual.
